# Diagnosis of Prenatal-Onset Achondrogenesis Type II by a Multidisciplinary Assessment: A Retrospective Study of 2 Cases

**DOI:** 10.1155/2019/7981767

**Published:** 2019-07-17

**Authors:** Wenbo Wang, Qichang Wu, Li Sun, Xiaohong Zhong, Yasong Xu, Xiaojian Xie, Zhiying Su

**Affiliations:** Prenatal Diagnosis Center, Women and Children's Hospital, School of Medicine, Xiamen University, Xiamen, Fujian Province 361000, China

## Abstract

**Aim:**

Achondrogenesis type II is a rare, lethal osteochondrodysplasia with considerable phenotypic heterogeneity. We describe our experience in diagnosing prenatal-onset achondrogenesis type II by a multidisciplinary assessment.

**Methods:**

Two cases of fetal achondrogenesis type II were analyzed retrospectively using prenatal ultrasound evaluation, postnatal radiographic diagnosis, and molecular genetic testing of* COL2A1*.

**Results:**

A causative mutation in the* COL2A1 *gene was found in both patients. Combined with postnatal radiographic examination, the final diagnosis of achondrogenesis type II was made.

**Conclusion:**

Our findings emphasize the importance of a multidisciplinary assessment for the definitive diagnosis of achondrogenesis type II, which is paramount for proper genetic counseling.

## 1. Introduction

Achondrogenesis is a lethal perinatal skeletal dysplasia with an incidence of 1 in 40000 live births; it is characterized by severe short-limbed dwarfism [1, 2]. Patients exhibit considerable clinical and radiographic heterogeneity; accordingly, achondrogenesis has been divided into two subgroups, i.e., type I (Parenti-Fraccaro) and type II (Langer-Saldino). Achondrogenesis type I exhibits autosomal recessive inheritance, whereas most type II cases are sporadic, resulting from new autosomal dominant mutations [3]. In particular, achondrogenesis type II is caused by structural mutations in collagen II and thus constitutes the severe end of the spectrum of collagen II chondrodysplasias [4].

Fetal achondrogenesis has significant variability in disease presentation; however, this variation is poorly understood, making diagnosis difficult. In this study, we describe our experience in diagnosing prenatal-onset achondrogenesis type II by a multidisciplinary assessment, including prenatal ultrasound evaluation, postnatal radiographic diagnosis, and molecular genetic analysis of* COL2A1*. These data may improve the detection of fetal achondrogenesis type II and provide an adequate basis for genetic counseling.

## 2. Subjects and Methods

### 2.1. Subject Population

The Prenatal Diagnosis Center of Xiamen Maternal and Child Health Care Hospital is a regional referral center for fetuses with suspected anomalies and/or genetic syndromes. It provides prenatal services for a considerable percentage of suspected anomalous pregnancies in the southwest area of Fujian of mainland China. In 2013–2015, two cases of severe fetal short-limb dwarfism detected by antenatal ultrasonography were referred to our center. The maternal age was 33 and 25 years old, respectively. The two Chinese women were in nonconsanguineous marriages and had a normal course of pregnancy; their family histories did not include reports of skeletal malformations. Because their antenatal sonographic features were associated with neonatal or infantile lethality, the families decided to terminate the pregnancies. In China, pregnancy can be terminated in any trimester if the fetus has severe malformations. Before the termination of pregnancy, cordocentesis was performed for fetal karyotyping and molecular analyses. Postmortem radiographic examinations were performed in all instances for definitive diagnosis. The study was approved by the Ethics Committee of Xiamen Maternal and Child Health Care Hospital (approval number 2015-62).

### 2.2. COL2A1 Gene Sequencing and Variant Detection

Genomic DNA was extracted using the QIAamp DNA Blood Midi Kit (Qiagen, Hilden, Germany) according to the manufacturer's standard procedure and fragmented using the Covaris LE220 (Woburn, MA, USA) to generate a paired-end library (200–250 bp). The library was enriched, followed by elution and post-capture amplification. Using the Agilent 2100 Bioanalyzer (Santa Clara, CA, USA) and ABI StepOne Real-Time PCR System (Waltham, MA, USA), the magnitude of enrichment was estimated. After quality control, the captured library was sequenced using Illumina HiSeq 2500 Analyzers (San Diego, CA, USA) for 90 cycles per read to generate paired-end reads. Image analysis, error estimation, and base calling were performed using Illumina Pipeline (version 1.3.4). After bioinformatics processing, the sequences were aligned to the human genome reference (hg19) using the BWA (Burrows Wheeler Aligner) Multi-Vision software package. SOAPsnp and SAMtools were used to detect single nucleotide variants and indels. All single nucleotide variants and indels were filtered and searched against multiple databases, including NCBI dbSNP, HapMap, the 1000 Human Genomes Project dataset, and the database of 100 Chinese healthy adults [5]. All mutations and potential pathogenic variants were validated using conventional Sanger sequencing methods. To predict the effect of missense variants, a subset of dbNSFP, which contains seven well-established in silico prediction programs (Scale-Invariant Feature Transform [SIFT], PolyPhen-2, LRT, MutationTaster, and PhyloP), was used.

## 3. Results

**Figure 1:**
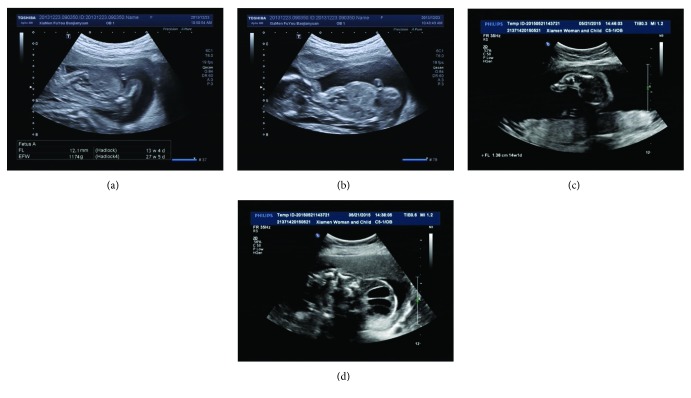
(a) Case 1: sonogram of femur length, which is extremely short. (b) Case 1: ultrasound scans showing narrow thorax and protuberant abdomen. (c) Case 2: sonogram of femur length, which is extremely short. (d) Case 2: ultrasound scans showing a large cystic hygroma.

**Figure 2:**
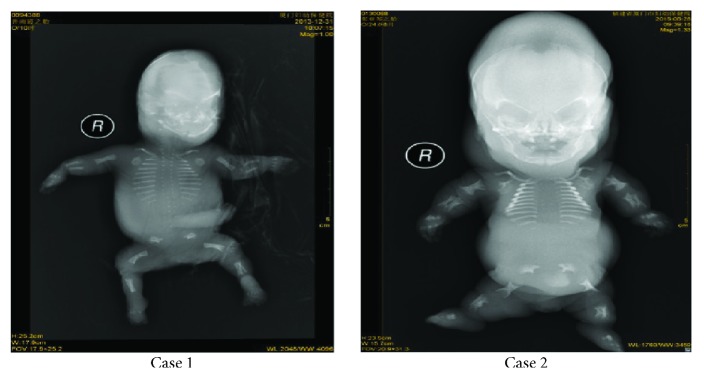
Radiography shows defective skull ossification; vertebral bodies unossified; short, cupped ribs, no fractures; iliac bone ossification limited to the upper part, giving a paraglider-like appearance; long bones, severely short; absence of ossification of all limbs.

Sonographic features of the two cases showed that the fetal femur length was extremely short and the fetuses had hydropic appearance (Figure 1). The postnatal radiographic examination of the two cases revealed fetal severely short long bones; defective skull ossification; vertebral bodies unossified; short, cupped ribs without fractures; and iliac bone ossification limited to the upper part, giving a paraglider-like appearance (Figure 2). These radiologic features are typical of achondrogenesis type II. The fetal karyotypes of the two fetuses were normal. The fetuses had distinct heterozygous* COL2A1 *mutations; the details of these mutations are shown in Table 1. The COL2A1 mutation of case 2 was in the collagen triple helix, but mutation of case 1 was in the carboxyl terminal propeptide region; the two causative mutations in COL2A1 have not been previously reported.

## 4. Discussion

Achondrogenesis is a rare, lethal short-limbed dwarfism. Traditionally, achondrogenesis is subclassified into type I (Parenti-Fraccaro), caused by recessive mutations of the diastrophic dysplasia sulfate transporter gene (*SLC26A2*), and type II (Langer-Saldino), caused by autosomal dominant mutations of the type II collagen gene COL2A1 [6]. Achondrogenesis is characterized by considerable phenotypic variability, including variation in prenatal sonographic features, radiographic findings, and molecular bases.

Recent advancements in prenatal ultrasonography have made it easy to detect fetal skeletal dysplasia, especially severe short-limb dwarfism. Lethal skeletal dysplasias can be accurately detected in the antenatal period; however, the diagnosis of specific skeletal dysplasias is challenging. Postnatal radiographic examination is typically used for distinguishing the different disorders. Among all skeletal dysplasias, achondrogenesis is characterized by the most severe limb reduction, and the defective cartilage produced by chondrocytes results in inadequate ossification [7]. Achondrogenesis type II is characterized by various degrees of calcification of the pelvis, skull, and spine, without ribs fractures [8]. In general, skeletal abnormalities are less severe in type II than in type I, but differentiating between these types is not possible based on radiographic features owing to the phenotypic overlap [9].

Over several decades, owing to the considerable phenotypic variability, achondrogenesis type I and II have been distinguished based on clinical, radiologic, and histopathological features [10]. In our experience with the prenatal sonographic diagnosis of achondrogenesis, the fetal femur length was the best biometric parameter to distinguish achondrogenesis from other skeletal dysplasias. By analyzing the constellation of findings, such as a narrow thorax, poor mineralization of the skull and vertebrae, polyhydramnios, a pseudohydropic appearance, and cystic hygroma, the determination of neonatal lethality and a differential diagnosis can be achieved. Our radiologic examination revealed severely shortened long bones, skull ossification, unossified vertebral bodies, and short, cupped ribs without fractures; iliac bone ossification was limited to the upper part, giving a paraglider-like appearance. With the assistance of a molecular genetic analysis to detect* COL2A1* mutations, the final diagnosis of achondrogenesis type II was definitively made. Therefore, we suggest that* COL2A1* mutations are a key feature for the recognition and diagnosis of achondrogenesis type II. Molecular genetic analysis of* COL2A1 *can be a diagnostic hallmark in achondrogenesis type II.

*COL2A1 *mutations give rise to a spectrum of phenotypes predominantly affecting cartilage and bone, from severe perinatally lethal disorders to milder conditions that are recognized in the postnatal period and childhood [11]. Achondrogenesis type II belongs to type II collagenopathies; it is a lethal chondrodystrophy due to the failure of cartilaginous matrix formation, and this phenotype may be associated with a defect in chondroitin sulfate or type II collagen synthesis. The* COL2A1* gene is located on 12q13.11 and contains 54 exons; it is a helical molecule with propeptide ends on either side. The backbone of type II collagen is the collagen triple helix characterized by a homotrimer of three collagen II chains. Achondrogenesis type II and hypochondrogenesis are both caused by heterozygous missense mutations in the collagen triple helix, resulting in the substitution of glycine with a bulkier amino acid [12]. This change distorts helix formation and/or stability and causes a dominant negative effect in cartilage.* COL2A1* mutations are universally private with no known mutational hot spots, but mutations in the carboxyl terminal propeptide region result in severe and lethal skeletal dysplasia [13]. However,* COL2A1* mutations are associated with substantial phenotypic variability. In our study, we described cases with distinct, previously unreported heterozygous type II collagen mutations. Very little is known about the mechanisms by which specific* COL2A1* mutations lead to particular phenotypes, but an assessment of the clinical significance of* COL2A1 *variants is critical to provide accurate counseling for affected families.

There has been substantial progress in the identification of the molecular defects responsible for skeletal dysplasias. Our results suggest that* COL2A1* mutations can be a diagnostic hallmark in prenatal-onset achondrogenesis type II, and the definitive diagnosis ideally requires a multidisciplinary assessment. With the discovery of COL2A1 gene variants as a cause of achondrogenesis type II, sequence analysis of the gene will add to the diagnostic process.

## Figures and Tables

**Table 1:** Mutations in *COL2A1* for the 2 cases.

Subject	Exon	cDNA	Protein	Software prediction	
				PolyPhen	SIFT
Case 1	EX53/CDS53	C.4231delC	p.Leu1411CysfsX24	Damaging	Damaging
Case 2	EX43/CDS43	C.2897G>C	p.Gly966Ala	Damaging	Damaging
